# Confessions of a Patient after Recovering from an Attack of Lunacy
*Annales d' Hygiène.


**Published:** 1850-07-01

**Authors:** 


### CONFESSIONS OF A PATIENT AFTER RECOVERING FROM AN ATTACK OF LUNACY.*

The narrative which we propose submitting to our readers, is from the person herself, who had been the subject of insanity.

The individual in question is a lady who had received a highly accomplished education. Her imagination was lively, her character was most marked in its disposition to conceive projects, and abandon them as soon as formed. She has been perfectly restored to health for several years. One day she was asked, if she had any recollection of her illness; if she knew how she had been affected by external objects, and what idea she had formed of the circumstances going on around her? Taking her personal experience as the grounds of her belief, she assured us, that, despite of the mental derangement, the insane remember the objects and the individuals who surround them during their most ungovernable excitement, even when they are incapable of distinguishing, from their rhapsodies, real objects and events, and that these recollections do not disappear after the cure. This fact led her to the belief that there were unknown reservoirs in the brain, where the impressions conveyed through the medium of the optic and auditory nerve are received and retained. She was convinced, she added, that, returning to the country where first her mind had wandered, not only did she recognise all the individuals who had been in communication with her at that period, but that she could even repeat the conversations carried on with those persons. For example, she had the most precise recollection of the order of the things in the room where she was confined, of the furniture of the apartment, and of the pictures and engravings which adorned the walls. On which, she remarked, in the treatment of lunatics, sufficient attention could not be paid to matters of that kind, as they frequently suggest to the patients the most distressing and painful thoughts.

This lady, possessed of a highly irritable temperament, and a most exalted imagination, conceived a misunderstanding at a moment when she was already the victim of disappointed hopes. The conjunction of these circumstances became the exciting cause of her mental affliction. She had in Holland claims to a large sum of money, but the date of her right was at a remote period, while another family, and with all appearance of justice, had made good their titles to the same possession. Advantageous offers, and the expectation of succeeding, by being present on the ground, urged her to proceed to Holland. After many useless plans, and after having seen all her efforts fail, she returned one day home with her feet very damp. The succeeding day she felt out of order, suffering much from cold feet, and pains of the head and throat. Instead of reposing in her bed, and promoting perspiration to recover her health, she sat at her desk to arrange a very long paper on her business, to which she devoted all her mind and means, so as to prove the justice of her claims. But notwithstanding the paper was written with great power, and she had presented the subject under every variety of aspect, it had no better success than the preceding memoirs. No answer was made to it; and when she called on the people to whom it had been transmitted, they always contrived to escape seeing her. Impatient, soured, and irritated at this cruel treatment, she had determined to return home, and had proposed leaving her lodgings, when she received a letter from her family, which induced her to protract her stay some time longer in Holland. The memorial which we have mentioned was the chief subject which engaged the disordered mind of this lady during the illness she had at that period. We now append the written detail which she gave of her feelings during her attack. Some few points in her history have been suppressed.

"During these transactions, I hired more retired apartments, and less dear. My landlord, a shoemaker, and all his family were worthy people, and obliging. I took them for Christians, though they were Portuguese Jews. When I was informed of that circumstance, I became painfully affected. I began to be under constant apprehension that they would rob me of my money. This fear increased to such an extent, as to deprive me of my rest. At last, I fancied that my host might some day make me swallow a narcotic draught, and assassinate me, along with my daughter, during the night, to get possession of my money. My suspicions received additional confirmation from the circumstance that these persons had prevailed on me to inscribe my name at the police-office as Madame *H. A.*, and not Madame *H. B.* Tortured by fear, for the period of eight days, I scarce slept for a few instants. My food was composed of eggs, fruits, and tea; and one day after having partaken of some bread which my landlady brought me, I was immediately attacked by a severe diarrhœa, and I had no more rest.

"My hostess explained the accident by a statement that the police, in order to prevent an epidemic with which the country was threatened, had directed the bakers to introduce into the bread designed for the lower orders, medicines which would act as a general purge.

"My body and my head broke down, weakened by the low diet, and by the continual watching. Fear carried them away. I felt my judgment going apace along with the power of reflection; and at last I was unable to draw from any given fact conclusions in accordance with the relations of that fact. The persons around me became still more fully suspected by me; and the end was, the loss of my reason.

"Two dreams, one of my daughter, the other about myself, occurring in the same night, brought my disease fully out. My daughter told me, that she had witnessed me throwing myself into the street from the third flat of a house in the town, and that I remained stretched on the pavement broken in pieces, and dead. We went to try and discover the house which she had seen in her dream; it was the Court of Judicature. As for my dream, it was that a man, bearing a purse, had entered the house of the Portuguese Jew, and had cut my throat. The day after I was busy washing some clothes, when raising my eyes I saw (and I was wide awake) a long knife passing over the ceiling of my room. Struck with alarm, I bade my daughter to be silent. In great haste I placed all my money in my work-bag, I closed my trunk, and hurried my daughter into the street, taking with me all my most important papers. I cannot say, whether some person had not, by way of joke, passed a knife through a slit in the ceiling, or whether it was not altogether a vision, the creation of my excited imagination. This, however, is undoubted, that I was quite awake, and in full possession of all my wits, when I saw the instrument of death. I had met shortly before, in descending the staircase, a man with a large purse under his arm, probably a barber. The appearance of that man deprived me of my self-possession; and once out of the Jew's house, reason completely deserted me. I then went to one of the body-guard. I addressed a young officer, and begged him with fervour to carry immediately to the king the packet of letters on me; but as he hesitated, and left me under the pretext of calling a superior officer, I hastened away from him, and went to the German Chancery, where I compelled the worthy keeper of the records, M. Z., to take my packet and preserve it for me. I also told him of my causes for alarm, and made him acquainted with the danger I dreaded. He took leave of me after having offered some common-place consolations, and I found myself again in the street. Here, however, everything was changed as far as regarded me. The city, so tranquil but a moment ago, was in the height of an insurrection. The regiment quartered in the garrison was Jewish. The prince royal and the king had been made prisoners and condemned to death. The enemy had broken ground at Schevelingen. The Asiatic hordes commanded by the Jews. Of what use could the gold be to me? I said to myself, and I returned to my landlord's door. I called his wife. I threw my money down on the work-table, advising her to begin a petty trade with it; and I concluded by a humble request for one louis, that I might return to Germany.

"The face of the poor Jewess must have actually been seen at the instant, when she received so unexpectedly a large gift, to conceive the astonishing effect it had on her countenance—it actually became purple. She could not divine how to explain the matter; but she concluded in offering me a piece of gold, and would have allowed me to go away without any further remark, had not her husband come in. He took a handful of the louis, and slipped them, almost without my consciousness, into my bag. The louis, however, were restored too late, from which cause I was led to believe the family highly honourable. Having in this manner, as I supposed, got rid of my money, my dread of being assassinated vanished, and I reasoned with tolerable precision for an insane person. I said to myself, the people would have killed you on account of your money; let them have it; they will countermand the assassin, and you may return home without any fear. I made this all clear to my daughter, and I took the road to Delft. I wished to pass the night in that town, and travel by the boat to Rotterdam, whence I would have proceeded to Munster by Arnheim and Emerich. I was desirous to see Madame H——, at Munster, and explain to her that it was a sacred duty she owed to her husband to recall him immediately from Holland, as he ran the hazard of being branded, as one individual had already experienced, who had put in his claims for a property.

"I had changed my louis at the banker L——, and I was already close by the gate of the city, when I saw a young Jewess following me; and though I had made different turns to avoid her, she nevertheless hung close on my footsteps. I then went up to her, and exclaimed, in a menacing tone, 'Accursed pagans! you have already crucified Christ, and this day you would vent your wrath on the Prince Royal!' The Jewess saved herself from this dreadful apostrophe, and from that moment I was fully satisfied that the prince, who was universally beloved, was in imminent danger. I then came in contact with an enclosed palisade. I asked what was the purpose of it? Being answered that it belonged to a Jew, I persuaded myself that it was the prison of the royal family. The absurd thought excited so much pain and sympathy in my heart, that I deserted my daughter, and desired with my nails, using all my force, to make an aperture in the enclosure, that I might save the prince, and bring him out along with me. Nothing could withdraw that fixed idea from my mind, which led me to the belief of war.

"This idea was further substantiated by two new visions, which existed nowhere but in my disordered brain. I saw then on the canal a little boat, with black sails and colours. My eldest daughter, whom I had left at C——, had taken refuge there, and was miserably clad. The boat, however, could not move, as the King of the Jews, under the penalty of death, had forbid any of the boatmen weighing anchor. That I might not betray her and let her understand that she was my daughter, I returned silently; and soon after I recognised the face of a young lady of H——, in full dress, coming out of a beautiful coach, and proceeding to an adjoining house. I followed this lady, to address her, but those whom I spoke to said that they had seen no one. In all haste, I then took the road to Delft, where I arrived at eight o'clock in the evening. I looked out for a respectable house for lodgings, but they would receive me nowhere. Finally, I was received into the house of Captain B——, whose lady was sick and confined to bed. Nevertheless, the people of the house showed a great interest for me, and treated me with great kindness and humanity. A new accession of fever came on, and a host of visions, more or less fantastical, all relating to the imprisonment of the prince royal, excited a furious delirium of the most extravagant nature, in consequence of which the persons with whom I resided carried me, in the course of the night, to another house. On the subsequent day a letter was despatched to the keeper of the records, M. Z.: he came to me in a closed carriage, and took me to an establishment at a distance from the street, where I was put under the care of an old servant of M. H. A physician was called in, and at the expiration of three weeks I was so far recovered, that my guardians no longer could trace my thoughts, though my ideas still clung to the same subject.

"After having left the house of M. B——, at Delft, I fell into a state of profound melancholy. I fancied myself to be in positions which only the extreme of madness can conceive. My recollections are by no means very clear of what occurred when we were at the hotel, where we had to pass three days; still, I have a floating idea of having conversed with different people, and that I answered different questions. I think, also, that when I went to bed, a great many people came to observe me, and they talked together about my condition, but all the rest was as a dream.

"The condition, however, in which I spent the first night seems worthy of attention. I thought myself abed, perfectly conscious, but totally unable to make any movement, in an immense abyss, in which I believed I had been buried alive, and had now awakened in the tomb, in the condition I was to live for all eternity, with the perfect consciousness of my condition, to reflect on myself. My mind, which, when awake a few hours previously, had been carried away by the most extravagant phrenzy, still enjoyed all its perceptions clear. I discussed with myself whether I deserved so stern a fate, and as I was unconscious of any crime done with premeditation, I concluded by supposing that this severity of punishment had been awarded to me because, though I had fulfilled my duties as much as lay in my power, I had yet neglected to do any good beyond my line of duty, &c. In other respects, I was in the same condition as a person affected with tetanus.

"I recovered myself, however, though I was in a state of extreme debility, not having sufficient strength almost to support the weight of my body. Scarcely was I awake ere I relapsed into my illusions. I began to scrutinize my room, that I might discover whether I had not fallen into the house of a merchant of souls (‘*Query*, Armi’). The burlesque motions with which I prosecuted this search would undoubtedly have provoked a smile in the most serious person, and at last I went into the chimney, reasoning thus with myself, that as it was made of stones, it could not be thrown down when the house was demolished. My fears were further augmented by the pictures which ornamented the walls. In that posture I awaited in trepidation the approach of the inmates of the house. A young girl appeared, who gave me some confidence, but when I saw my old landlady enter, my emotion could not be concealed; and lastly, when two keepers were brought into the room, who were not to leave me, my wrath was fired anew, and I broke a window that I might escape.

"After some time I was permitted to go to the garden; the open air soothed me, and yet everything around me was a source of illusion to me. The houses around the garden seemed to me to be prisons filled with prisoners. I fancied the kitchen of my landlady, in which a large pot was boiling, the place where the prisoners were put to the torture. The water of the pot in which they were going to throw me, I thought was boiling oil. Full of that notion, I tore the sleeve off my daughter's robe, desirous to retain it that she might not incur the hazard of being boiled alive.

"All this receives its explanation in the condition of a phrenetic lunatic, all whose actions are influenced by so many dreadful fancies; so it is always with me, that it is impossible to alleviate, even a little, those agonies, except I am completely enlarged from them.

For if I had been shut up on that day, or even bound down by chains, either fright would have stopped the flow of the blood in my veins, or it would have circulated with such intense rapidity, that, with undoubted certainty, all the arteries would have burst in my brain. Most luckily I was left in the garden, though a violent storm was approaching. I felt myself very well when my keepers were forced to retire by the rain under the protection of the alley of the house, leaving me at full liberty to contemplate the rising storm. But how different was that storm from that I had seen before, and those I have witnessed since. The clouds which rolled up from the horizon appeared to me to be the billows of the deep, rising o'er the banks of the Schevelingen to the skies, fighting in the air together over my head; while a flotilla of the enemy, on the margin of the river, carried on a deadly combat against the inhabitants. The last hour had struck for the prosperity of Holland. I did not hear any thunder; I did not witness any lightning; but I perceived the explosion of a hundred blazes of fire, the cannonade, ceaseless, reverberated in my ears: from which we may infer, with all certainty, that the ear and the eye of the insane amplify and enlarge whatever is heard or seen.

"The same remark occurred to me afterwards. As my symptoms appeared better, my linen and my property were restored to me. I took them out of my trunk and arranged them on my table. I was struck with their great number, and even with the appearance of a cloth and towels, which, however, I had left behind at C——. But this joy did not continue long; and when the following day I again examined my linen, a great many objects seemed a-wanting, which I had fancied to have had in my hands the previous evening; so much so, that I supposed I had been robbed. I did not, however, communicate my suspicions to any one.

"These two circumstances justify me in affirming that the lunatic fancies he sees and hears objects which have no real existence. But what I am now going to mention proves the important influence of an individual, opportunely seen, in giving a proper degree of assurance to the sick person, for the earliest symptoms of my recovery take their date from the day when I saw, amongst a great many others, a form that particularly caught my attention.

"I cannot well say whether it was the second or third day several persons came to talk with me in the garden, but I was extremely insolent to every one, even to Captain B——, to whom I owe my life. At the end two men opened the gate, and looked on my side of the garden; one was dressed in a deep blue overcoat, and he almost immediately withdrew; the other was dressed in very beautiful uniform; he also retired. After that a young man of a very good expression entered, having all the outward appearances of perfect health; he spoke to me in French, and I answered him in the same language. I took this person for the Prince Royal, and the bandage fell from my eyes. I felt myself all of a sudden in great confusion for appearing before the prince in a *costume* so unsuited for the occasion. I was surprised that he was still alive, and as he appeared in perfect health, the anxieties I had experienced on his account, conceiving that the enemy, which had beleagured the country, had made him suffer great torment, all vanished in a moment. I felt myself as if inspired with a new life, and from that hour the visions of horror were no more.

"It will be easily understood that this young person was not the prince, though he was a little like him. What an infinity of good would be conferred on the lunatic could his thoughts be anticipated, and scenes of a nature to affect him favourably be brought before him. Had permission been given me to leave that day, I assuredly would have committed nothing either that was ridiculous or attended with injury to any one. But there were still more cruel trials in reserve for me, from which I was not to escape until I had gone through the ordeal of three additional days' illness.

"A coach was ordered, in which M. Z., the keeper of the records, conveyed me to La Haye, where I was placed in a house near the castle. I then had a difference with M. Z., as we did not leave the town by the same gate we had entered. I attempted to show him that he had mistaken the road, and I felt much offended in perceiving that he, with a smile on his lips, continued the same route, without paying any attention to my observations. When we stopped, this irritation was further increased on perceiving a child looking at us. I said to it that it deserved the rod, which caused it to run away. As I ascended the stairs I counted the steps; and I was again thrown into distress on getting to my room, when I saw that the door could not be locked from within.

"My alarm, however, became extreme, when I firmly believed that I thought I recognised in the person of my nurse an individual whom I had seen hanged some time before at La Haye, along with another criminal, and whom accordingly I took for a spirit. In the solitude of night, I perceive myself alone in company with this person, full of the most agonizing apprehensions. I would not allow the shutters to be closed at nightfall; and as, when I thought I had seen the prince, I had no longer any dread of war, I was fully persuaded that our soldiers had been victorious, so this idea stirred up in my breast the fears of being assassinated. When the pump was worked in the yard I fancied that they were going to throw the water up into my room, and I looked every moment to see it rushing in. Noticing three nails in my room, I supposed that they intended to hang us on them, myself, my daughter, and my nurse, because the latter had been condemned to death.

"Resting on my couch one evening, but quite awake, I watched every step of the nurse with my eyes, as I thought her a spirit; the candle ran, but I did not observe the tallow flow from *that* candle, but from a hole in the wall, whence it was discharged in an enormous quantity, resembling a furious torrent which has burst through its banks, so that I screamed aloud, and pretended that they were going to suffocate me. The incident made me suspect that they had the intention to poison the atmosphere, and ever from that moment I

constantly experienced a disagreeable though sweet smell. All the viands offered to me had that taste. I thought that the meat they brought was human flesh, and insisted on the idea that they desired to poison me. Since my complete restoration to health I have discovered, in one of my walks, a poisonous plant, which had the disagreeable odour I allude to.

"The circumstance I have referred to, of the tallow running down the wall, is a convincing proof to me that persons labouring under disorder of the mental faculties, perceive objects which have no real existence, and that the sight of particular matters produces, spontaneously, images in the eye of the diseased person.

"Even at a later period, when I was improving, I still saw Dr. T——; then my brother-in-law; I heard the voice of my sister, as also another voice, which, speaking to me by my name, bade me 'lay down the petition.'

"I often requested of my keepers to have my clothes, my papers, and my money; but they answered me that they were to be kept till my husband appeared, who ought to come and inquire for me. On several occasions I objected to this arrangement (pleading the expense it would be attended with) to interest the persons who detained me to permit me to travel alone; this, however, they would not accede to, though I had become much more calm. Several dreadful dreams broke in on this state of tranquillity, tallying, however, very appositely with my condition. There I was, in the realms of Pluto below, which I examined with a remarkable degree of firmness and self-possession. I saw, moreover, the *aqua tolena* prepared. I had read an account of this horrible torture, the frightful details of which were all reproduced in the dream, and my children were the unhappy victims of this barbarity of the Italiani. I would rather suffer in reality every kind of imaginable torture, than again experience that horrible dream. On being awakened I found that I had been dreaming, but still one uneasy idea succeeded another, and the last of the kind was on my return, after having been in a 'diligence.'

"We might be almost persuaded to conclude from these facts, that every visible object should be withdrawn from the eye of the lunatic; but if what I witnessed gave rise to misinterpretation on my part, those things which were concealed from me excited still more extraordinary conjectures.

"I converted the office in the house into a chamber where the torture was performed; every time I heard a packet sealed I thought it was the *coup-de-grace* of some unfortunate wretch. An old apartment, always closed, containing old records, and full of armories, was the charnel-house, and the armorer represented the coffins. I firmly believed that the story above me was a conservatory for the remains of those who had been assassinated, until one day, finding the door open, and all being still in the house, I went up quietly myself to ascertain how far my painful suspicions were well founded. Great, then, was my happiness, when, instead of bones, skeletons, and carcases, I saw nothing but torn old waste paper. My curiosity was wound up to the highest pitch, and yet I had not courage to touch one of the leaves. I opened a window which looked into the royal garden; the windows of the apartment of the king also commanded a view of the garden. I noticed at one of the windows a tall lady in white robes; the moment I saw her she rose from her chair somewhat hastily, and I supposed she was the princess. From that moment all my fantastical notions were centered in that princess, as I thought she was detained as a prisoner in that room.

"I looked then by the windows in the front of the house, where I was, and I noticed a range of buildings which surrounded the castle in the form of a circle. It would be interesting to ascertain whether, from the windows of that roof, the view which I describe here can be enjoyed in perfection, to determine whether my senses were not under the sway of an illusion, when I saw a crowd of magnificent mansions all around in that quarter. The front buildings could be perceived from my bed-room. I saw distinctly a small earthen pipe, which passed by the chimney of the house nearest the court of the castle; and it was not a long mental operation for me to conclude that the tube of that pipe was the only mode of the air having access to the house. So I likewise inferred that all the individuals who entered the house would be suffocated.

"On the day of my husband arriving, and in his presence, my whole system underwent a special change. Instead of feeling a satisfaction that I had in him a protector, I was harassed by the idea of being considered insane by him, and being placed under the control of a person whom I distrusted. Under the influence of that fear, I exercised all my self-control, that he might not suspect my insanity, though I was still far from being in full possession of my wits. I also adopted the precaution to procure secretly a strong dose of rhubarb. I swallowed it all at once, and felt myself much better after. I had done so formerly with benefit.

"Some days after the arrival of my husband, we began our arrangements to return home. We secured places in the diligence, though we would have done better by hiring a carriage, as we had to pay for three seats. We were then fairly on our road, and the shocks and jolts of the wretched vehicle in which we travelled were of no small service in restoring my addled brain. I soon found that my reason was restored.

"We arrived for the night at a town beyond A——, where we were to stay till the morning. We had a bedroom, but there was no lock to it. When my husband had undressed and gone to bed, I noticed that he had left his pantaloons near the door, which was ajar, and I was afraid lest he had left money in his pockets. I searched them, and found, to my great delight, thirty-two double Louis, of those which I had taken with me; and, in addition, the sum of two hundred *reichsthaler*, in single Louis's. I immediately concealed the thirty-two double Louis's in my clothes, intending, if my husband did not adopt better arrangements for our journey, to start alone on foot, and manage the gold coins myself. This money, which I had worked hard for in my early life, and which I had recovered, imparted to me a new spirit, so that, from that instant, I felt that I had entered on a new life. My fears all vanished, and everything about me appeared under a new light. Desirous to give my husband some little annoyance, as a punishment for his want of prudence, I placed in his bed the money which belonged to him, retaining my own. The following morning his alarm was great when he found his pockets empty, though his pantaloons were on the chair he had put them on the previous evening. I comforted him, restoring to him the money, and told him that his manner of travelling, though it was highly extravagant, was not the more pleasant on that account; that I would not contribute any more to the general expenses, but pay only for those of myself and my daughter. Notwithstanding my remonstrance, as he persisted in travelling by *diligence*, I left him in a village, and proceeded alone as far as the gates of Westphalia. I should undoubtedly have lost my way, had it not been for an incident which has much the appearance of the marvellous.

"Arriving at a place where three roads cross, I was going to follow that which would have brought me back to the point whence I had started, when I noticed the tracks of a man who had probably conveyed corn to the town of Minden : a sack had burst, and a considerable amount of the corn had escaped. My head was still feeble, and I had an explanation ready for this adventure: I conjectured then, and very luckily this time, that this corn had been spread on the road to enable me to escape from the labyrinth in which I was involved. I followed the marks with perfect confidence, and treading steadily on the corn, I passed over roads almost impracticable, and through several villages, getting finally into the high road, where I met the diligence, which, taking that route, had made a long circuit: there I joined my husband and my daughter.

"At Minden, I took the arrangements for the continuance of our journey into my own hands, and hired a private carriage for ourselves. Notwithstanding this, the most trivial circumstance suggested erroneous fancies; but as I was in a state of perfect liberty, I examined very attentively the subjects which had awakened surprise in my breast, and I gradually became conscious of my errors. I still recollect several of these very singular visions.

"At the period of which I now speak, I was in no way uneasy as to my own fate, or that of my family, but I was distressed by a feeling of sympathy for the Jews, discomfited, as I thought, in Holland, and scattered in the woods in the neighbourhood of C——, where they were perishing of hunger and cold, along with their wives and children. I daily resorted to the woods and deposited bread and money, particularly near the cross-roads.

"Two regiments passing through the city at the same time, had a coffin in their escort: this circumstance affected me with alarm, for I thought that their king was in the coffin. To convince myself of the truth of the circumstance, I ran across the garden to meet the procession; but the body had disappeared, and afterwards I understood that the coffin was tenantless. I called a young soldier, who was following the regiment at a distance; I made several questions to him on the subject, but he did not answer me, but went away without saying one word, to a hillock covered with verdure and thorns; he there made a hole in the midst of the thorns with his cane. He still declined answering me, when I asked him whether a king had not been buried on one of the banks of the Rhine? I was soon, however, satisfied that the silent soldier was a spirit, which idea made me exceedingly uncomfortable.

"Fear, probably, and the stormy season to which I had been constantly exposed, again disturbed the harmony of my intellectual powers. From that day, as soon as I arrived in the country, I observed on the summit of all the mountains which circumscribe the horizon, machines which appeared to me to be telegraphs, and I fancied, at the conclusion, that the enemy, after having cut a canal, had beat back the Prussian army as far as the Rhine, driving it into the deep, and that they were anxious to preserve the vessels and the corpses of the parties so destroyed, as trophies of their victory. This idea excited in my mind a determined hatred against the barbarous men capable of so atrocious a deed; and to show that I could not be blamed for being a party to its execution, the strange notion came into my head to send some loaves and a bottle of brandy to several detachments of recruits on their route through the town:" they took the brandy, and handed the loaves over to the poor.

Since this lady returned to her native city, her visions, though not exhibited by outward signs, as she has now acquired sufficient self-control to conceal them from the world, are still frequently renewed. She retained the notion for a long time, that the Jews had resolved to destroy the Christians. She also saluted with much courtesy and humility all the Jewesses; if they were clothed in rags, she addressed them in terms of extreme politeness, offered them her kind offices, and endeavoured to comfort them. She sometimes gave the poor Jews a piece of money, in which she conceived there was some particular virtue.

At last, she gave up this notion, as she became daily convinced that her apprehensions were altogether chimerical; but she adopted a notion exactly the reverse. This contrast is often noticed in the dreams of the insane. She fancied that a great number of Jews were encamped in an immense forest behind a mountain in the vicinity of the town where she lived—that the government kept them prisoners there, and watched them, and that they were condemned to perish a wretched death by hunger. Actuated by sympathy for those unfortunate beings, and indignant at the cruel measures enforced against them, she ventured several times out near the forest, and placed at different parts by the way-side, all kinds of food, such as loaves, fruits, eggs, &c., so that these unhappy creatures might pick them up, and that some of them at least might escape from the dreadful death to which they had been doomed.

(*To be continued.*)

